# Effect of Momordica charantia fruit extract on vascular complication in type 1 diabetic rats

**DOI:** 10.17179/excli2014-539

**Published:** 2015-01-30

**Authors:** Razif Abas, Faizah Othman, Zar Chi Thent

**Affiliations:** 1 Department of Anatomy, Universiti Kebangsaan Malaysia, Jalan Yaacob Latif, 56000 Cheras, Malaysia; 2 Department of Human Anatomy, Faculty of Medicine and Health Science, Universiti Putra Malaysia, 43400 Serdang, Selangor

**Keywords:** Momordica charantia, fruit extract, diabetes mellitus, vascular complication

## Abstract

Diabetes mellitus is one of the risk factors in the development of vascular complications. Decreased nitric oxide (NO) production and increased lipid peroxidation in diabetes mellitus are the dominant exaggerating factors. *Mormodica charantia* (*MC*) was proven to be useful in improving diabetes mellitus and its complications. In the present study, a total of 40 male Sprague-Dawley rats were used. Diabetes was induced by a single dose (50 mg/kg) of streptozotocin (STZ), intramuscularly. Following 4 weeks of STZ induction, the animals were equally divided into five groups (n = 8); Control group (Ctrl), control group treated with *MC* (Ctrl-*MC*), diabetic untreated group (DM-Ctrl), diabetic group treated with *MC* (DM-*MC*) and diabetic group treated with metformin 150 g/kg (DM-Met). Oral administration of the* MC* fruit extract (1.5 g/kg) was continued for 28 days. DM-*MC* group showed a significant decrease (P < 0.05) in blood pressure, total cholesterol and triglyceride levels compared to the DM-Ctrl group. Aortic tissue NO level was significantly increased and malondialdehyde level was decreased in the DM-*MC* group. Immunohistochemical staining showed an increase in eNOS expression in the endothelial lining of the DM-*MC* group. Similarly, morphological deterioration of the aortic tissues was reverted to normal. In summary, treatment with the *MC* fruit extract exerted the significant vasculoprotective effect in the type 1 diabetic rat model.

## Introduction

Diabetes Mellitus (DM) is a chronic oxidative stress disorder which occurs due to the insulin deficiency or resistance. Chronic hyperglycaemia causes glycation of the advanced glycoproteins and leads to the development of macro and microvascular complications (Virdi et al., 2003[39]). Increase free radical formation in the diabetic state promotes the increase in oxidative stress and decrease in anti-oxidant level (Sathishsekar and Subramaniam, 2005[33]). This mechanism enhances the lipotoxicity and endothelial dysfunction in certain tissues (Lin et al., 2011[24]; Stratton et al., 2000[36]). Hyperglycaemia induces a large number of alterations in the vascular tissue that accelerate the vascular wall lesions. Exposure of endothelial cells with higher glucose levels leads to a reduction in the nitric oxide (NO) level, a potent endothelium-derived vasodilator in the major blood vessels. The production of NO depends on the endothelial nitric oxide synthase (eNOS) enzyme which is involved in the regulation of vascular tone (Giugliano et al., 1996[16]). Therefore, increased oxidative stress is responsible for the development of macrovascular complications in DM.

A wide array of dietary food was reported to control oxidative stress and lipotoxicity in DM. *Momordica charantia* (*MC*), belongs to the family of *Cucurbitaceae*. This dietary food has been widely used in South East Asia. It is known as bitter gourd or bitter melon in English and *‘Peria’* in local Malaysia (Grover and Yadav, 2004[17]). The fruit of *MC* is enriched with steroidal saponins known as charantins, insulin-like peptides and alkaloids. It contains several amino acids, which reduce the blood glucose level thereby preventing DM. Previous studies reported *MC* to possess hypoglycaemic, anti-hyperglycaemic (Raman and Lau 1996[31]), hypotensive (Ojewole et al., 2006[27]) and lipid lowering properties (Ali et al., 1993[1]). A recent pharmacological study with aqueous extract of *MC* showed a significant reduction in the blood glucose level compared to chloroform extract (Karunanayake et al., 1990[20]).

The fruit extract of *MC* was tested in various tissues of diabetic animal model and the active compounds from the extract were discussed in past studies (Lii et al., 2009[23]; Pycnogenolu and Dysfunkciu, 2013[29]). However, the effect of the *MC* fruit extract on the vascular tissues in DM is still remained obscure. Hence, the present study was designed to observe the protective effect of the *MC* fruit extract on vascular complications in type 1 DM. Such investigations will open the door for the potential therapeutic approaches of the *MC* fruit extract for future studies.

## Materials and Methods

### Preparation of MC fruit aqueous extract

The *MC* fruits (7 kg) were purchased from the local market, Selayang, Malaysia. The entire plant was identified by a Botanist from Universiti Kebangsaan Malaysia (UKMB 40067). The sun-dried fruits of *MC* were cut into small pieces, powdered and extracted with distilled water between 75-80 °C for 3 hours. The extracts were evaporated at 80 °C freezer and stored at 4 °C until use. In this study, the oral dosage of 1.5 g/kg body weight was used for the treatment of animals (Waheed et al., 2008[40]).

### Animals

We used forty (n = 40), male Sprague-Dawley rats weighing 250 - 350 g obtained from Laboratory Animal Resource Unit, Faculty of Medicine, UKM, Malaysia. Prior ethical approval was taken before starting the experiment. All efforts were made to minimize animal suffering and to reduce the number of animals used. The rats were acclimatized for 1 week. During the experiment, the rats were kept in an individual plastic cage, maintained under standard environmental conditions, temperature at 25-28 °C (12-h light/dark cycle) with free access to tap water and rat pellets. 

### Induction of diabetes

Following overnight fasting, the experimental group of rats received a single intramuscular injection of a dose of 50 mg/kg body weight of streptozotocin (STZ) (Lenzen, 2008[22]) whereas control groups received 0.9 % normal saline. Three days following STZ induction, the fasting blood glucose level was measured in all the rats. A fasting blood glucose level of more than 8 mmol/L was considered as diabetic (Thent et al., 2012[37]). Following 4 weeks of STZ induction, the animals were equally divided (n = 8) into the control group (Ctrl) and the control group treated with *MC* extract (Ctrl-*MC*), diabetic control group (DM-Ctrl), the diabetic group treated with *MC* extract (DM-*MC*) and the diabetic group treated with 150 mg/kg metformin (DM-Met). The dose of metformin was adopted from a previous study (Scheen, 2013[34]). The treatment was continued for 28 days. 

### Measurement of blood pressure

Blood pressure was monitored by non-invasive tail-cuff CODA system. The rats were exposed under the sunlight for 10 minutes before the measurement of the blood pressure. The systolic, diastolic and mean arterial blood pressures were measured at baseline, pre-treatment and post-treatment following *MC* administration. An average was taken following 5 readings at each measurement (Fokkema et al., 1995[14]).

### Measurement of fasting serum lipid levels (FSL)

Total cholesterol, HDL cholesterol, LDL cholesterol and Triglycerides were measured in plasma of individual rats from each group at baseline, pre-treatment and post-treatment periods. The blood was collected from a retro-orbital vein. The collected whole blood was then sent to Pathlab & Clinical laboratory (M) Sdn Bhd Malaysia for further analysis.

### Collection of tissues

At the end of the study, i.e. at 28 days following *MC* treatment, the rats were sacrificed with the overdose of diethyl ether. The thoracic part of aortic tissues were excised, cleaned and the adhered connective tissues were removed. Lipid peroxidation and nitric oxide level were measured by homogenizing the thoracic aorta. For microscopic study, the aortic tissue was incised into 2 mm^3^ diameters and then fixed with 10 % formalin.

### Determination of malondialdehyde level

Malondialdehyde (MDA) level was estimated by the double heating method (Draper and Hadley, 1989[12]). The principle of the method is the spectrophotometric measurement of the colour generated by the reaction of thiobarbituric acid (TBA) with MDA. For this purpose, 2.5 ml of the I-1trichloroacetic acid solution was added to 0.5 ml supernatant in each centrifuge tube and the tubes were placed in hot water bath for 15 minutes. Then, the tubes were centrifuged again and the supernatant was added to the TBA solution in a test tube. The concentration of MDA was calculated by the absorbance coefficient of the MDA-TBA complex and was expressed as nanomoles per gram of protein.

### Determination of nitric oxide level

Aortic tissue samples were homogenized and centrifuged. Then, Premix was prepared by mixing 50 µL 1.0 nM standard and 450 µL distilled water. A 100 µL of each homogenised sample was added to the separate wells. Then, Working Reagent (WR) was prepared and mixed in each sample and standard tube and was incubated for 10 minutes at 60 °C. Briefly, it was then centrifuged and transferred into the wells. Absorbance was read at 500 – 570 nm (peak 540 nm) (Guo et al., 2000[18]).

### Immunohistochemical analysis

After perfusion and paraffin embedding, the slides were deparaffinized and rehydrated in Xylene series. The slides were rinsed until ready for staining. The primary antibody was incubated and following 24 hours of incubation, the secondary antibody was added. Slides were tested using PBS as negative control. The eNOS expressions were observed by the double blinded fashion (Cosentino and Luscher, 1999[9]).

### Light microscopy

The thoracic part of aortic tissues were processed for fixation, dehydration, clearing, infiltration, impregnation and embedding. The tissues from individual rats were fixed with 10 % formalin. The tissues were dehydrated with alcohol series and cleaned with xylene in automatic tissue processor unit. The tissues were paraffinized and the blocks were cut by using the microtome (5 µm thickness). Eventually, thickness of the tunica intima (TI) and tunica media (TM) were measured in each group under image Analyzer software (Moraes-Teixera et al., 2010[25]). The sections were then, stained with haemotoxylin and eosin; alcian blue and orcinol stainings.

### Statistical analysis

The data were presented as the mean ± standard error of mean (SEM). Statistical analysis was carried out by using ANOVA followed by Bonferroni and/or Tukey’s post-hoc test. The value of P < 0.05 was considered to be significant. All statistical analysis was performed using the SPSS statistical package version 21.0 (SPSS Inc., Chicago, USA).

## Results

### Blood pressure

**Figure 1:**
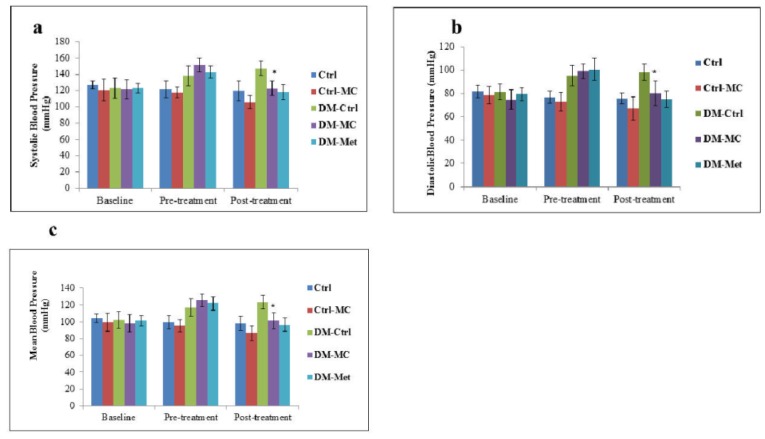
Effect of *MC* fruit extract on the blood pressure of Sprague-Dawley rats * Indicates a significant decrease (P < 0.05) in a(SBP), b(DBP) and c(MBP) mmHg of the DM-*MC* group compared to the DM-Ctrl group.

The baseline, pre-treatment and post-treatment SBP, DBP and MBP were measured in each rat. At the end of pre-treatment, a significant increase (P < 0.05) in SBP, DBP and MBP were observed in all the experimental diabetic groups. Following 4 weeks of treatment (i.e. post-treatment period), there was a significant decrease (P < 0.05) in SBP, DBP and MBP in the DM*-MC* group, i.e. 122.5 ± 8.8 mmHg, 80.17 ± 10.21 mmHg and 101.33 ± 9.5 mmHg, respectively, compared to the DM-Ctrl group, I. e. 147.67 ± 8.98 mmHg, 98.33 ± 6.86 mmHg and 123 ± 7.92 mmHg, respectively. These findings were compatible with the findings in DM-Met group. No significant changes were observed between the non-diabetic groups (Figure 1(Fig. 1)).

### Fasting serum lipid level

**Figure 2:**
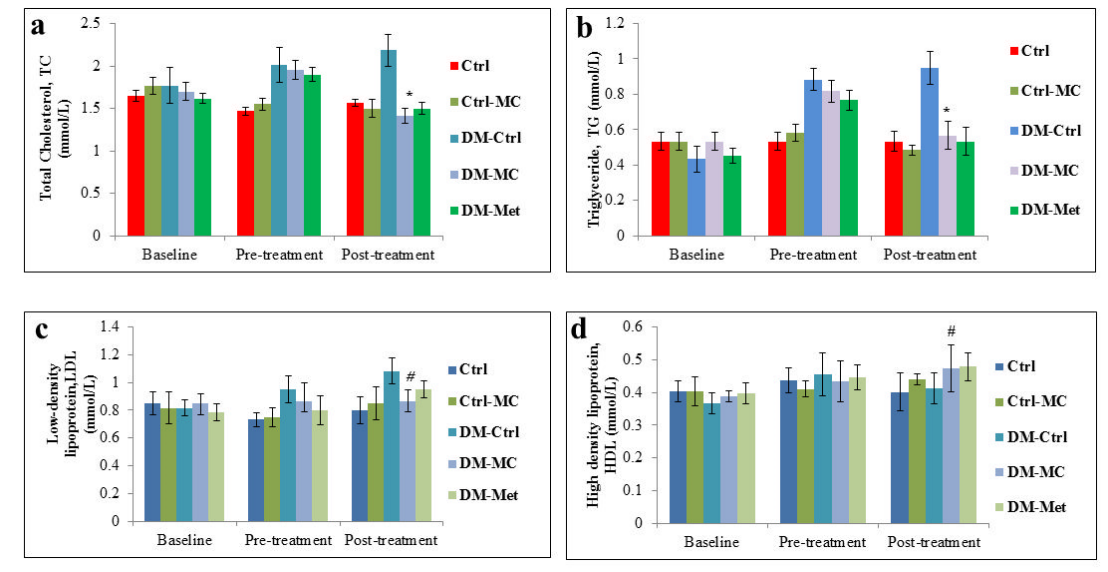
Effect of *MC* fruit extract on the fasting serum lipid profile of Sprague-Dawley rats. *Indicates a significant decrease (P<0.05) in a(TC) and b(TG) levels of the DM-MC group compared to the DM-Ctrl group. # Indicates no significant difference (P>0.05) in c(LDL) and d(HDL) levels.

At the end of the pre-treatment period, there was an increase in the fasting serum lipid level in all the experimental diabetic groups. Following 4 weeks of treatment, there was a significant decrease (P < 0.05) TC and TG levels in the DM-*MC* group (1.42 ± 0.08 mmol/L and 0.57 ± 0.08 mmol/L, respectively) compared to the DM-Ctrl group (2.18 ± 0.19 mmol/L and 0.95 ± 0.09 mmol/L, respectively). Similar findings were observed in DM-Met group. However, no changes were observed with regard to the levels of HDL and LDL in all the groups in the post treatment period (Figure 2(Fig. 2)).

### Aortic tissue MDA level

At the end of the study, aortic tissues were homogenized to analyse the MDA level. DM-*MC* group showed a significant decrease (P < 0.05) in the MDA level (6.06 ± 0.86 nmol/mg) compared to the DM-Ctrl group (19.73 ± 1.57 nmol/mg). Furthermore, DM-Met group (9.13 ± 1.87 nmol/mg) showed slightly decreased (P < 0.05) MDA level. However, the Ctrl-*MC* group showed no significant changes in MDA level compared to the Ctrl group (Table 1(Tab. 1)).

### Aortic tissue Nitric Oxide level

Nitric oxide (NO) level was analysed in the aortic tissues of individual rats. DM-*MC* group showed a significant increase (P < 0.05) in the NO level (1329.58 ± 104.61 µM) compared to the DM-Ctrl group (785.83 ± 99.53 µM). The result was found to be correspondence with DM-Met group (1196.25 ± 64.68 µM). However, Ctrl-*MC* group showed no significant increase in NO level compared to the Ctrl group (Table 1(Tab. 1)).

### eNOS expression in aortic tissue

**Figure 3:**
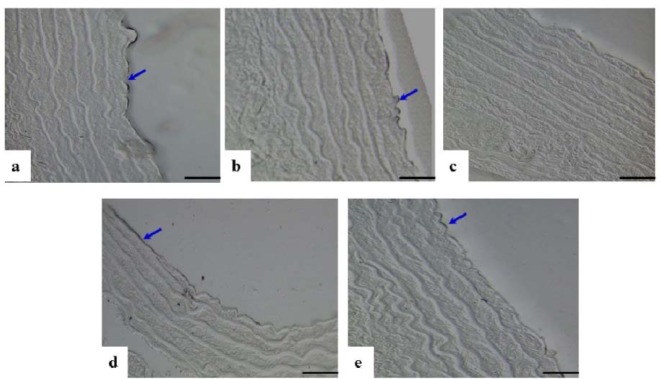
Photomicrograph showing cross sections of the thoracic aorta under immunohistochemical staining for eNOS expression. a(Ctrl), b(Ctrl-*MC*), c(DM-Ctrl), d(DM-*MC*), e(DM-Met). Note the increase eNOS expression in endothelial lining (stained black, blue arrows) in c(DM-Ctrl). LM X 200

Under immunohistochemical staining, endothelial nitric oxide synthase (eNOS) expression was shown as black in colour in the endothelial lining of the tunica intima. Findings showed little or no eNOS expression in the DM-Ctrl group. The thickness of TM also prominently increased. There was reduced elasticity of the elastic fibres in the DM-Ctrl group. However, the aortic tissue in the DM-*MC* group showed an increase in the eNOS expression, less TM thickness and more elasticity compared to the DM-Ctrl group. Furthermore, DM-Met group showed increased expression of eNOS in the endothelial lining. No changes in eNOS expression were observed in the non-diabetic groups (Figure 3(Fig. 3)).

### Histomorphometric analysis of thickness of TI and TM

The histomorphometric analysis of the thickness tunica intima (TI) and tunica media (TM) were observed by using the image Analyzer software. Four measurements per image were obtained at 0°, 90°, 180° and 270° and the mean was taken (Table 2(Tab. 2)). It was observed that there were no significant differences in the thickness of TI in all groups of rats. However, the thickness of TM was found to be significantly decreased in the DM-*MC* group (89.31 ± 1.65 µm) compared to the DM-Ctrl group (121.72 ± 2.95 µm). Similar findings were observed in the DM-Met group.

### Histological findings

**Figure 4:**
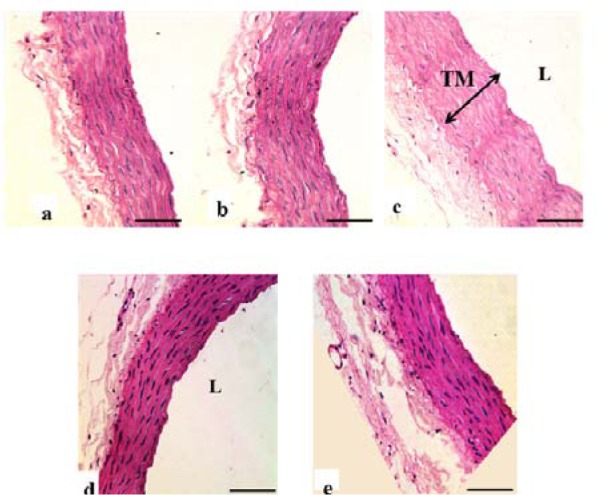
Photomicrograph showing transverse sections of the thoracic aorta under H&E staining. a(Ctrl), b(Ctrl-*MC*), c(DM-Ctrl), d(DM-*MC*), e(DM-Met). Note the relative thickness of the TM in c(DM-Ctrl) with double headed arrow. TM= Tunica Media, L= Lumen. LM X 200

**Figure 5:**
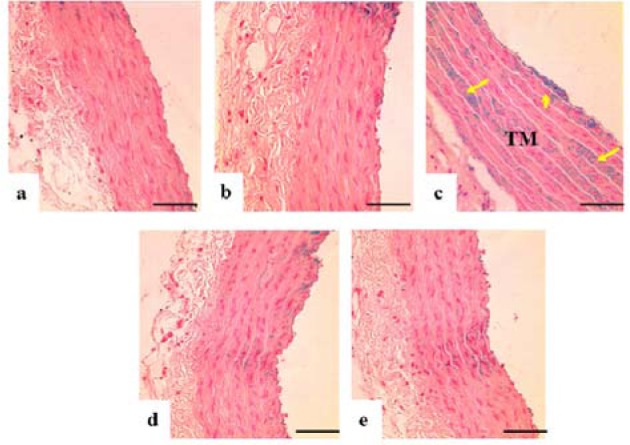
Photomicrograph showing transverse sections of the thoracic aorta under Alcian blue staining. a(Ctrl), b(Ctrl-*MC*), c(DM-Ctrl), d(DM-*MC*), e(DM-Met). Note the presence of acid mucopolysaccharides (stained blue, yellow arrows) in c(DM-Ctrl). TM= Tunica Media, LM X 200

**Figure 6:**
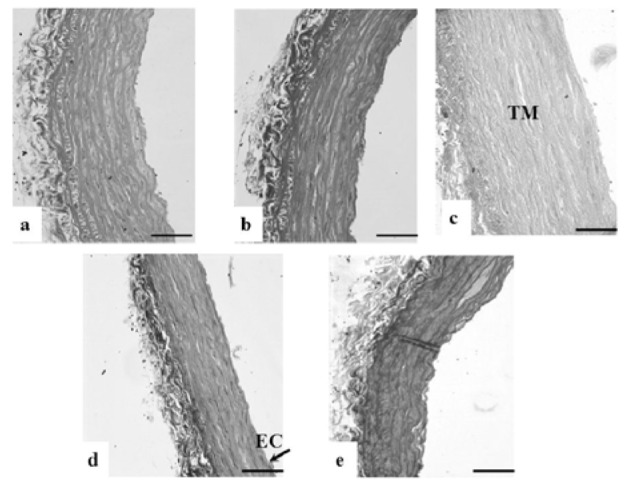
Photomicrograph showing transverse sections of the thoracic aorta under Orcinol staining. a(Ctrl), b(Ctrl-*MC*), c(DM-Ctrl), d(DM-*MC*), e(DM-Met). Note the reduced tortuosity of elastic fibres in c(DM-Ctrl). TM= Tunica Media, EC= Endothelial cell, L= Lumen. LM X 200

Loss of endothelial cells in TI, disrupted elastic lamellae and increased thickness were observed in the DM-Ctrl group. However, DM-*MC* group showed less pathological changes compared to the DM-Ctrl group. The presence of endothelial cells in TI and reduced TM thickness was observed in the DM-*MC* group under H&E staining. Similar features were observed in the DM-Met group (Figure 4(Fig. 4)). Alcian blue staining is a special staining used to stain the connective tissue deposition, especially acid mucopolysaccharides. The increased acid mucopolysaccharides deposition was observed in the DM-Ctrl group which appeared blue in colour. However, less acid mucopolysaccharides deposition with blue staining in the DM-*MC* group was observed compared to the DM-Ctrl group (Figure 5(Fig. 5)). Orcinol staining is used to delineate the deposition of elastic fibres in the large arteries. The histological findings of thoracic aorta in the DM-Ctrl group showed decrease in tortuosity of the elastic fibres. However, these degenerative changes were found to be improved in the DM-*MC* group. Tortuous elastic fibres with intact endothelial cells was observed in the DM-*MC* group. Similar findings were observed in the DM-Met group (Figure 6(Fig. 6)).

## Discussion

In the present study, a significant increase (P < 0.05) in SBP, DBP and MBP in the DM-Ctrl group was observed following post-treatment period. It was reported that STZ-induced DM rats have a high risk of increase in the blood pressure (Browne et al., 2003[7]). SBP plays a crucial role in DM, by increasing the pumping effect of the left ventricle. It was stated that the increase in collagen deposition and myocardial fibrosis leads to develop the systolic hypertension (Boudina and Abel, 2007[6]). In an another study, it was mentioned that chronic hyperglycaemia without any treatment leads to increase DBP (Ojewole et al., 2006[27]). Therefore, an increase in both SBP and DBP, leads to an increase in MBP which was observed in the experimental diabetic rats. The blood pressure in the DM-*MC* group was found to be significantly decreased compared to the DM-Ctrl group (Figure 1(Fig. 1)). The exact hypotensive mechanisms of the *MC* extract for lowering blood pressure, still remains unclear. It can be explained that the active compound, leptin, present in the *MC* fruit extract attenuates the blood pressure. Leptin proved to reduce the blood pressure by increasing the anti-oxidant and nitric oxide efficiency (Sharma et al., 1996[35]). No significant difference in the blood pressure was noted in the Ctrl-*MC* group. 

Besides the blood pressure, the fasting serum lipid (FSL) profile of the individual rats was also examined in the present study. Research reports mentioned the diabetic patients to have a high risk of developing dyslipidaemia (Al-Neaimy, 2008[2]). Non-enzymatic reaction between the lipoproteins and hyperglycaemia leads to the overproduction of advanced glycosylation end products (AGEs), which accelerates the hyperlipidaemia in a diabetic state (Aronson and Rayfield, 2002[3]). In the present study, it was noted that there was a significant increase (P < 0.05) in total cholesterol (TC) and triglyceride (TG) in all the diabetic rats compared to the control rats during the pre-treatment period. Traditionally, normal mice and rats have not been ideal models for cardiovascular research since the animals have very low levels of LDL and high levels of HDL (Gajda et al., 2007[15]). Therefore, no significant changes in LDL and HDL were observed in all the groups of rats, throughout the study. Following 28 days of treatment, DM-*MC* group showed a significant decrease (P < 0.05) in TC and TG level compared to the DM-Ctrl group (Figure 2(Fig. 2)). The findings agreed with the previous study which reported a decrease in TC and TG levels in Wistar rats following treatment with the MC fruit extract (Fernandes et al., 2007[13]). It was documented in the early studies that *MC* fruit extract breaks down the hepatic enzymes involved in glucose and lipid metabolism, to reduce the serum lipid level (Wohaieb and Godin, 1987[41]; Bailey and Day, 1989[4]). However, purified active fractions of the* MC* fruit extract are yet to be tested for its hypolipidaemic effects. 

Increased lipid peroxidation plays a key role in the pathogenesis of vascular complication in the diabetic state (Ogura et al., 2006[26]). The increased MDA level is associated with an increase in the free radical production in the diabetic state. Several studies were conducted on peroxidative stress-induced atherosclerosis (Prasad, 2005[28]). In the present study, increased aortic tissue MDA level was found in the DM-Ctrl group. In DM-*MC* group, a significant decrease (P < 0.05) in the aortic tissues MDA level was observed compared to the DM-Ctrl group (Table 1(Tab. 1)). Earlier study showed the effect of* MC* fruit extract on the plasma MDA level in alloxan-induced diabetic rats (Tripathi and Chandra 2009[38]). In the presence of anti-oxidant compounds (e.g. plant phenols), *MC* fruit extract protects membrane lipid peroxidation (Bhoomi et al., 2013[5]).

Nitric oxide (NO), a potent vasodilator, inhibits leukocyte adhesion, smooth muscle proliferation and plaque instability due to the presence of anti-thrombotic property (De Dios et al., 2007[11]). In the present study, the DM-Ctrl group showed significantly reduced (P < 0.05) NO level. It was postulated that, reduced bioavailability of L-arginine, the precursor of NO, is an important factor contributing to the development of vascular complication in type 1 DM rats (Crea et al., 1997[10]). DM-*MC* group showed increased NO level compared to the DM-Ctrl group (Table 1(Tab. 1)). This may be due to the presence of the charantin compound in the *MC* fruit extract which produces lipopolysaccharide (LPS)-induced NO (Lii et al., 2009[23]). 

The enzyme nitric oxide synthase (eNOS), regulates the endothelial function in the vascular wall. In type 1 DM, the deficient insulin decreased the vascular relaxation through eNOS activity in the endothelial cells (Yan et al., 2006[42]; Quintela et al., 2012[30]). In the present study, the DM-Ctrl group showed less eNOS expression in the endothelial lining of the tunica TI under immunohistochemical staining. However, increased eNOS expression was observed in the thoracic aorta of the DM-*MC* group (Figure 3(Fig. 3)). It is believed that the anti-oxidant effect of *MC* phenolic and flavonoid compounds play a major role in the vasculoprotective mechanism in the diabetic state. Besides anti-oxidant and glycemic control, *MC* plays an important role in the protection of vascular endothelium against oxidative stress induced by ROS and nitrogen species (Pycnogenolu and Dysfunkciu, 2013[29]). 

In the present study, the morphological changes in thoracic aorta were analysed under the light microscope. The thickness of TI and TM of the thoracic aorta were measured (Table 2(Tab. 2)). It was found out that, following 8 weeks of STZ induction, the DM-Ctrl group showed increase in the TM thickness (Figure 4(Fig. 4)). A previous study explained that the increase in the thickness of TM was due to the increased proliferation of smooth muscle cells in DM (Rosenfield and Ross, 1990[32]). Under Alcian blue staining, more acid mucopolysaccharide deposition was shown in the aortic tissue of the DM-Ctrl group (Figure 5(Fig. 5)). An earlier clinical study showed that abnormal carbohydrate-protein substances in DM leads to the formation of acid mucoplysaccharides in the large blood vessel (Budin et al., 2009[8]). Moreover, it was reported that elastin and collagen content are altered in the arterial wall of DM patients as well as in the experimentally-induced diabetic animals. Orcinol staining was used to identify the features of the elastic fibres. In the DM-Ctrl group, there was less deposition of elastic fibres with reduced tortuosity in the vascular wall (Figure 6(Fig. 6)). In the DM-*MC* group, less abnormality was observed when viewed under the light microscope. The thickness of TM was found to be reduced significantly (Table 2(Tab. 2)). It was believed that *MC *has a potent effect in improving the development of vascular wall damage. It can be explained that the alteration in glycogen metabolism following administration with the *MC* fruit extract reduced the proteoglycans by reducing the sugar contents (Jayasooriya et al., 2000[19]). Therefore, DM-*MC* group showed less acid mucopolysaccharide depositions in the aortic tissue under Alcian blue staining compared to the DM-Ctrl group. The increased tortuosity of elastic fibres compared to the DM-Ctrl group under Orcinol staining might be due to the presence of anti-atherogenic and anti-lipidaemic properties in the *MC* fruit extract (Komolafe et al., 2013[21]).

In the present study, DM-Met group was used as a positive control group as metformin is known as the standard oral hypoglycaemic agent for the treatment of diabetes mellitus. The results achieved from DM-*MC* group was compared to the DM-Met group. Interestingly, the present findings were in accordance with the DM-Met group. With regard to the aortic tissue MDA level, DM-Met group did not show any significant positive effect. It may be due to the deficient antioxidant compounds present in the synthetic agent like metformin. 

From the present findings, it was observed that the administration with *MC* fruit extract for 28 days improved the vascular complication by decreasing the blood pressure, serum total cholesterol, triglyceride levels, aortic tissue MDA level and increasing aortic tissue NO level in type 1 diabetic rat model. These results were confirmed by the tissue histomorphometery, immunohistochemistry (eNOS expression) and histological analysis. In addition, the results of the *MC* fruit extract were in accordance with an oral hypoglycaemic agent, metformin. Our results are a key to establish the scientific foundation of *MC* fruit in improving the diabetic vascular complication. Further investigations in determining the mechanism of action of the *MC* fruit extract in the treatment of vascular complications arising due to diabetes mellitus are required. 

## Acknowledgements

This work was financially supported by Faculty of Medicine, Universiti Kebangsaan Malaysia (Grant no. FF-2013-422). The authors wish to acknowledge Prof. Dr. Srijit Das for his expert guidance towards this study.

## Figures and Tables

**Table 1:**
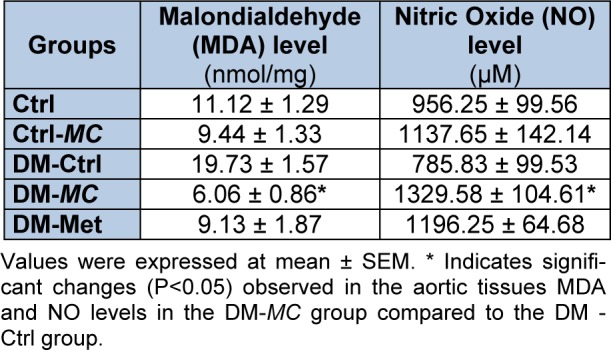
Effect of *MC* fruit extract on MDA and Nitric Oxide levels in the aortic tissues

**Table 2:**
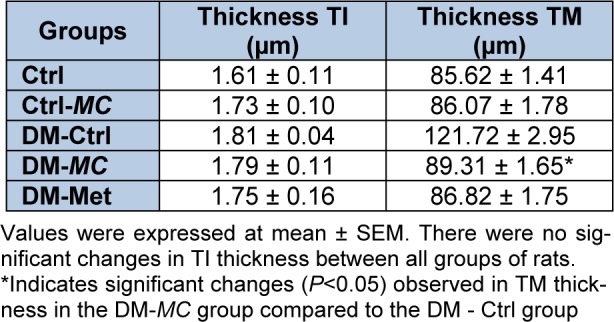
Effect of *MC* fruit extract on thickness of Tunica Intima (TI) and Tunica Media (TM) in the aortic tissues
